# Fragmentation and Isomerization Pathways of Natural and Synthetic Cannabinoids Studied via Higher Collisional Energy Dissociation Profiles

**DOI:** 10.3390/molecules30030717

**Published:** 2025-02-05

**Authors:** Kgato P. Selwe, Ambar S. A. Shaikh, Kelechi O. Uleanya, Caroline E. H. Dessent

**Affiliations:** Department of Chemistry, University of York, Heslington, York YO10 5DD, UK; kgato.selwe@york.ac.uk (K.P.S.); ass594@york.ac.uk (A.S.A.S.); kelechi.uleanya@york.ac.uk (K.O.U.)

**Keywords:** cannabinoids, synthetic cannabinoids, illicit drugs, high-resolution mass spectrometry, protomers, tautomers

## Abstract

Cannabinoid molecules are the family of molecules that bind to the cannabinoid receptors (CB1 and CB2) of the human body and cause changes in numerous biological functions including motor coordination, emotion, and pain reception. Cannabinoids occur either naturally in the Cannabis Sativa plant or can be produced synthetically in the laboratory. The need for accurate analytical methods for analyzing cannabinoid molecules is of considerable current importance due to demands for detecting illegal cannabinoids and for monitoring the manufacture of popular, non-illegal cannabinoid products. Mass spectrometry has been shown to be an optimum technique for identifying cannabinoids. In this work, we perform Higher Collisional Dissociation (HCD) mass spectrometric measurements on an Orbitrap Fusion Tribrid Mass Spectrometer to measure the collision-energy-dependent molecular fragmentation pathways of a group of key cannabinoids and their metabolites (cannabidiol, Δ9-Tetrahydrocannabinol, 11-Hydroxy-Δ9-tetrahydrocannabinol, 11-nor-9-Carboxy-Δ9-tetrahydrocannabinol, cannabidiolic acid, tetrahydrocannabinolic acid), along with two synthetic cannabinoids (JWH-018 and MDMB-FUBINACA). This is the first time that cannabinoid molecules have been studied using energy-resolved HCD methods. We identified a number of common, primary fragmentation pathways, including loss of water, loss of other small neutral molecule units (e.g., butene), and rupture of the central C-C bond that links the aromatic and alkyl ring groups. Quantum chemical calculations are presented to provide insights into preferred protonation sites and to characterize isomerization of protonated open-ring cannabinoids (e.g., [CBDA + H]^+^) into closed-ring analogues (e.g., [THCA + H]^+^). A key result to emerge from our study is that energy-resolved HCD measurements are particularly valuable in identifying isomerization, since the isobaric pairs of molecular ions studied here (e.g., [CBDA + H]^+^ and [THCA + H]^+^) are associated with identical HCD profiles indicating that isomerization of one structure into the other has occurred during the electrospray–mass spectrometry process. This is an important result as it will have general applicability to other tautomeric ions and thus demonstrates the application of energy-resolved HCD as a tool for identifying tautomerization proclivity.

## 1. Introduction

Cannabinoids are a class of molecules that bind to the CB1 and CB2 receptors in the human body. They can be found naturally (endocannabinoids) in the Cannabis Sativa plant (phytocannabinoids) or can be made in the lab (synthetic cannabinoids) [1]. This class of molecules produce a broad range of ecotoxicological effects and, as a result, have been studied extensively over recent years [2]. As an example, the main psychoactive compound found within cannabis plants is Δ9-tetrahydrocannabinol (THC), which is acknowledged to be the most consumed drug of abuse worldwide [3]. Cannabidiol (CBD), on the other hand, has been shown to possess medicinal properties for a broad range of ailments, and its use is therefore increasing globally [4]. Due to their potency, synthetic cannabinoids have been labeled as new psychoactive substances (NPSs) or “designer drugs” and are predominantly used recreationally [5]. NPSs are currently the largest group of substances monitored in Europe by the European Monitoring Centre for Drugs and Drug Addiction (EMCDDA) with 245 unique compounds reported in 2022 [6].

Over recent years, public opinion on the use of cannabinoids has become more favorable, as is evident in changes in legalization and concomitant increased use [7,8,9,10]. Increasing human consumption of cannabinoids inevitably results in increased concentrations of cannabinoids and their metabolites in household wastewater [11]. Inefficient treatment methods of wastewater treatment plants (WWTPs), underground leakages and sewer overflows can all result in transfer of primary source cannabinoids (and their metabolites) to the wider environment [12]. For example, 11-nor-9-carboxy-∆9-THC (THC-COOH), the main urinary metabolite of compounds containing THC, has been detected in both treated and untreated wastewater, and even in tap water [13]. All of these considerations mean that the analytical detection of cannabinoids and their metabolites should be an important part of water monitoring activities.

The need for advanced analytical techniques for accurately identifying cannabinoid family molecules is also currently being driven by the increased commercial production of non-illegal cannabinoid-based products, such as cosmetics and beverages which generally contain cannabidiol (CBD) [14]. CBD readily transforms into related compounds [14], so it is essential for such products to be analytically monitored both during production and also as a function of storage time and conditions. The further development of analytical techniques for accurately characterizing cannabinoid molecules is therefore highly desirable and numerous efforts towards this goal are underway [15,16,17].

Tandem mass spectrometry has proven to be a highly valuable technique for identifying cannabinoids, especially when used with a soft ionization technique such as electrospray ionization (ESI) [18,19,20]. One of the important aspects of a number of common cannabinoids and their metabolites is that they exist in tautomeric forms, which can make them problematic to identify by routine CID [21]. An important example is given by the cannabidiol (CBD) and Δ9-tetrahydrocannabinol (THC) pair [22]. For such systems, it is worth investigating whether collisional-energy-resolved profiles may be a better tool for identifying whether a selected precursor ion is associated with a unique geometric structure or two isomeric structures. While there have been numerous CID studies of cannabinoids, to date, there have been no collision-energy-resolved fragmentation studies.

**Scheme 1:**
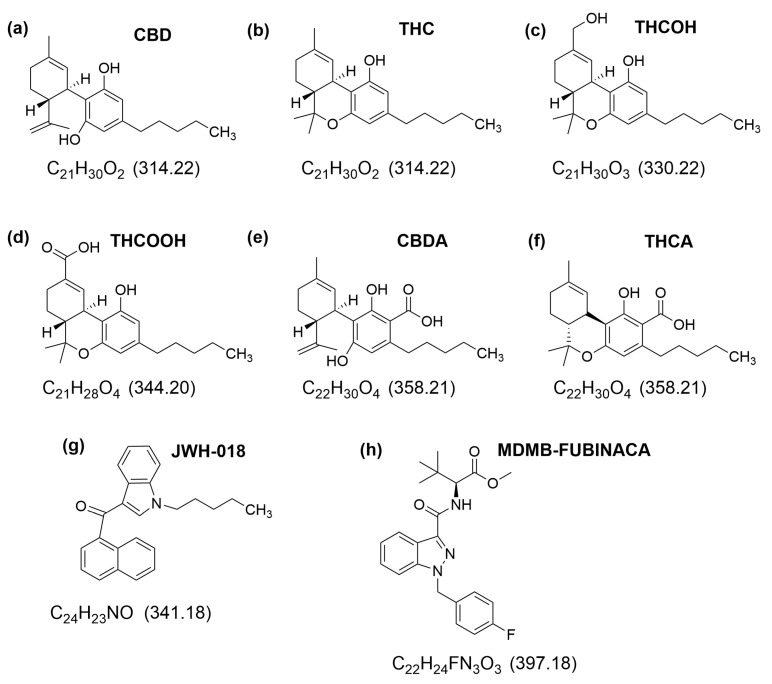
Structures, chemical formulae and exact masses (parenthesis) of the eight cannabinoids studied in this work, namely (**a**) cannabidiol (CBD), (**b**) Δ9-tetrahydrocannabinol (THC), (**c**) 11-hydroxy-Δ9-tetrahydrocannabinol (THCOH), (**d**) 11-nor-9-carboxy-Δ9-tetrahydrocannabinol (THCOOH), (**e**) cannabidiolic acid (CBDA), (**f**) tetrahydrocannabinolic acid (THCA), (**g**) JWH-018, and (**h**) MDMB-FUBINACA.

In this work, we perform the first Higher Collisional Dissociation (HCD) measurements to map the collision-energy-dependent molecular fragmentation pathways of a group of cannabinoids and their metabolites. The cannabinoids selected for study are shown in Scheme 1 and are studied here as their protonated forms following soft electrospray ionization. Compared with traditional, ion-trap-based collision-induced dissociation (CID), HCD provides ion beam CID and tandem MS with detection of fragment ions at high resolution in an Orbitrap mass analyzer [23]. Importantly, it allows for the identification of both primary molecular fragments which are produced at modest collisional energies, and also secondary fragment ions produced at higher collision energies [24], with continuous acquisition across an extended collisional energy range. In previous studies where we applied HCD mass spectrometry to molecular ions which can exist as isomers, we were able to observe small differences in HCD fragmentation patterns which are associated with precursor ions adopting different geometric structures [25,26]. This indicates that fragmentation-energy-resolved HCD measurements may be useful for identifying tautomeric forms of cannabinoid ions and motivates our current investigation. In addition to the experimental work performed, we supplemented the HCD measurements with quantum chemical calculations to provide a direct insight into the geometric structural preferences of a pair of cannabinoids where tautomerization can occur. Our experiments also allow us to identify common fragmentation pathways for this group of molecules that will be useful for predicting breakdown patterns for other natural and synthetic cannabinoids [27].

## 2. Results

### 2.1. HCD Fragmentation

**Figure 1:**
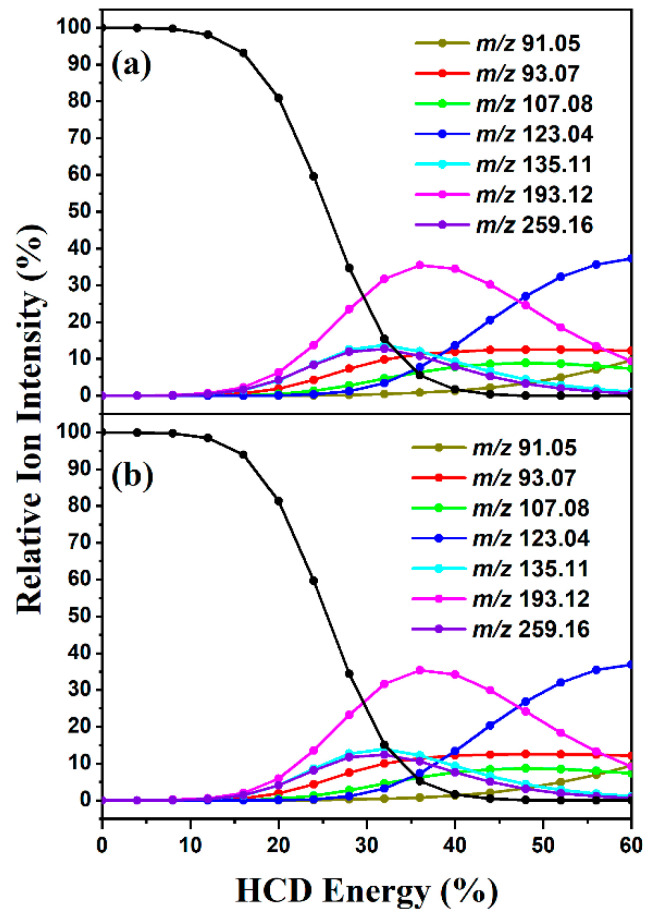
Precursor ion dissociation curve along with fragment ion production curves for (**a**) [CBD + H]^+^ (*m*/*z* 315.23) and (**b**) [THC + H]^+^ (*m*/*z* 315.23) displayed as a function of HCD energy. The solid line is a three-point adjacent average of the data points (the black line corresponds to the precursor ion).

Figure 1a displays the HCD curves for [CBD + H]^+^, illustrating that the precursor ion (*m*/*z* 315.23) has a relatively low threshold for fragmentation, with a dissociation onset around 8%. The *m*/*z* 193.12 fragment ion is the most intense fragment, displaying a production profile that peaks at 36% HCD energy. Two other lower-intensity (~12% relative ion intensity) primary fragments are observed with *m*/*z* 135.11 and 259.16, with profiles that peak slightly lower at 32% HCD energy. The intensity of these primary ions decreases as the HCD energy increases further (i.e., >40%). Two other primary fragments (*m*/*z* 93.07 and 107.08) are produced with similar dissociation energy onsets, but their profiles plateau above 32% HCD energy. Lastly, two secondary fragments, *m*/*z* 123.04 (major) and 91.05 (minor), are observed with onsets above 25% HCD energy and intensities which increase towards higher collisional energies. Secondary fragments are identifiable as fragments that have onsets at relatively high HCD energy, with their intensity increasing as the intensity of primary fragments decreases.

**Figure 2:**
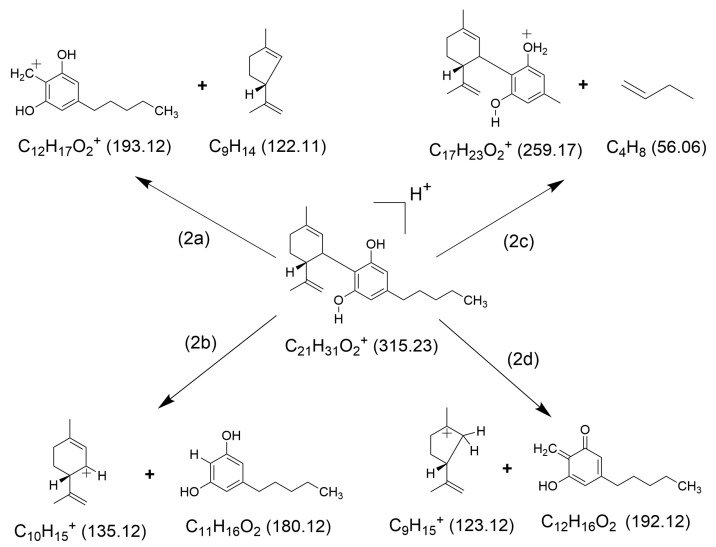
Suggested structures of the major ionic fragments and neutral counter-fragments (2a)–(2d) observed in HCD fragmentation of [CBD + H]^+^ (exact masses are given in parentheses). The pathways presented here follow those suggested by Broecker and Pragst [28].

Suggested geometric structures for the fragments associated with the various Collisional Dissociation pathways of [CBD + H]^+^ are shown in Figure 2, with the fragments following the assignments of the previous lower-energy CID study of Broecker and Pragst [22]. The major HCD fragment at modest collision energies, *m*/*z* 193.12, is associated with a rupture of the aromatic ring–cyclohexenyl ring central bond followed by a ring-size reduction of the cyclohexenyl ring to a pentenyl ring combined with a hydrogen shift (2a). This pathway is similar to the complementary (albeit more minor) one associated with the *m*/*z* 123.04 fragment ion (2d). The *m*/*z* 135.11 fragment is also formed by cleavage of the bond between the aromatic ring and cyclohexenyl ring, again accompanied by a hydrogen shift (2b). Finally, the *m*/*z* 259.16 fragment is associated with a β-cleavage of the pentyl group with elimination of a neutral butene molecule (2c). Interestingly, we do not observe the *m*/*z* 181.12 fragment (associated with the same C-C fragmentation as the *m*/*z* 135.11 fragment but with the charge on the opposite molecular fragment) that was observed by Broecker and Pragst, suggesting that the fragmentation pathway to *m*/*z* 181.12 is associated with a different thermal ensemble of the starting precursor ions.

Figure 1b displays the HCD fragmentation results for [THC + H]^+^, which is isobaric with [CBD + H]^+^. The similarity between the energy-dependent fragmentation curves for [THC + H]^+^ and [CBD + H]^+^ is striking: the dissociation onset for [THC + H]^+^ closely mirrors that for [CBD + H]^+^, identical fragments are produced from both ions, and the fragment profiles as a function of collision energy are identical within the experimental error. The similarity in the HCD fragmentation behavior of [CBD + H]^+^ and [THC + H]^+^ is consistent with previous mass spectrometry work on these systems. Broecker and Pragst observed isomerization of the two molecular ions via in-source hydrogen/deuterium experiments with hybrid quadrupole-time-of-flight mass spectrometry [22]. They concluded that [THC + H]^+^ likely rearranged to [CBD + H]^+^, and therefore, positive ion mode ESI-MS/MS of THC and CBD was not sufficient to unambiguously discriminate between these cannabinoids. This conclusion aligned with an earlier suggestion that an acid-catalyzed isomerization of CBD and THC can occur in solution. The identical fragmentation pathways and, in particular, the exactly identical HCD energy-dependent production profiles of the fragments from [THC + H]^+^ and [CBD + H]^+^ observed here provide strong evidence to support these earlier interpretations and suggest that one of these systems tautomerizes to the other, either in the electrospray solution or during the ionization process.

**Figure 3:**
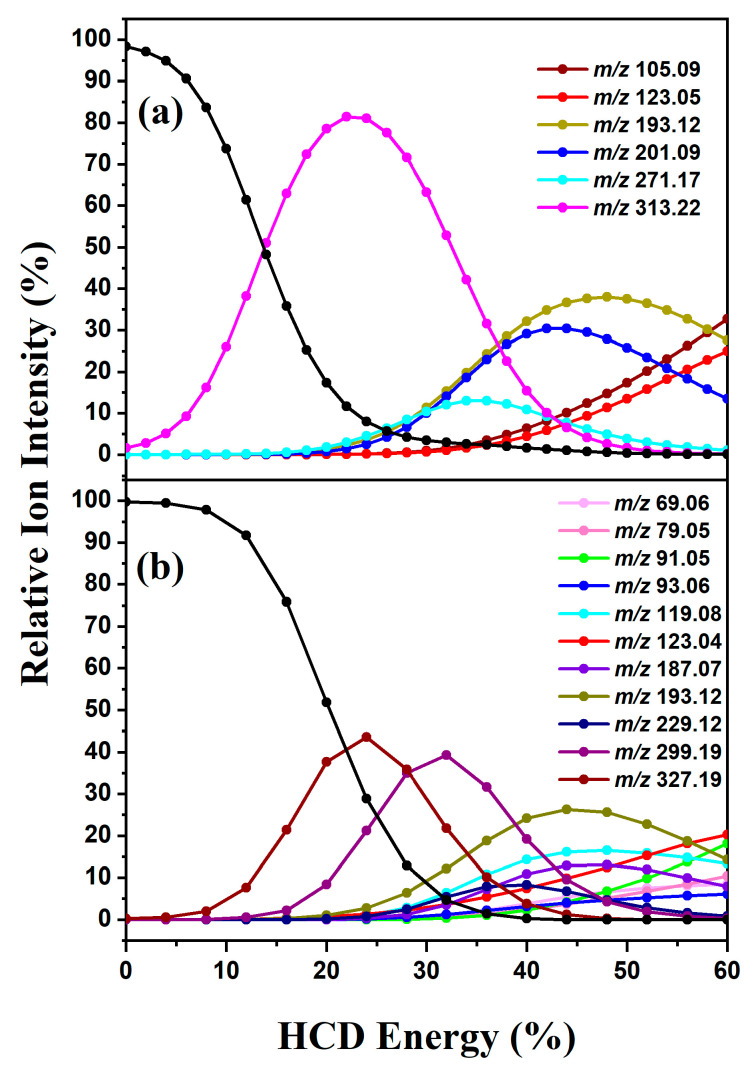
Precursor ion dissociation curve along with fragment ion production curves for (**a**) [THCOH + H]^+^ (*m*/*z* 331.21) and (**b**) [THCOOH + H]^+^ (*m*/*z* 345.20) displayed as a function of HCD energy. The solid line is a three-point adjacent average of the data points (the black line corresponds to the precursor ion).

Figure 3 displays the HCD curves for [THCOH + H]^+^ and [THC-OOH + H]^+^, along with their respective fragment ion production curves. THCOH and THCOOH are the main urinary metabolites produced upon consumption of THC, and are often used as environmental biomarkers for marijuana use [2,28]. [THCOH + H]^+^ can be seen to fragment readily at low HCD energy (Figure 3a), with an onset for fragmentation from 1 to 2% HCD energy. This reveals that the precursor ion is metastable, with a strong propensity to fragment. The initial fragmentation of [THCOH + H]^+^ can be seen to be linked to production of the *m*/*z* 313.22 fragment ion which is associated with facile loss of water from the precursor ion. Production of *m*/*z* 313.22 peaks at 22% HCD energy and then decreases at higher collision energies. A pathway associated with production of *m*/*z* 271.17 opens above 17% HCD energy, along with two additional fragmentation channels that produce the ions, *m*/*z* 201.09, and *m*/*z* 193.12 with very similar production profiles (these fragment ions are likely associated with loss of a water unit from *m*/*z* 201.09 to form *m*/*z* 193.12). At higher energies (>40% HCD), two lower-mass secondary fragments (*m*/*z* 123.05 and *m*/*z* 105.09) are produced with increasing intensity.

**Figure 4:**
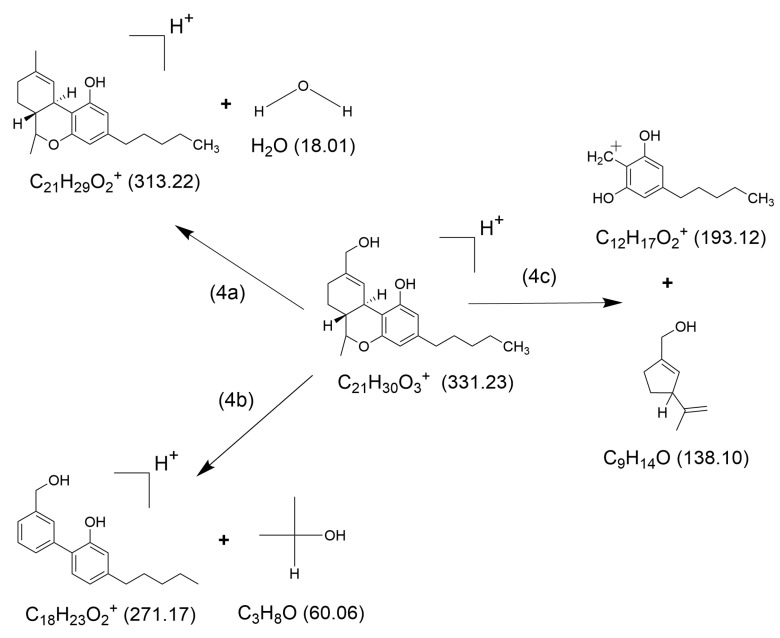
Suggested structures of the major ionic fragments and the neutral counter-fragments (4a)–(4c) observed in HCD fragmentation of [THCOH + H]^+^. Exact masses are given in parentheses.

Figure 4 presents suggested fragmentation pathways for the major fragment ion observed from HCD of [THCOH + H]^+^. Pathway (4a) is the dominant low-energy fragmentation pathway associated with ejection of water from the precursor ion. Production of the *m*/*z* 217.17 fragment ion (4b) can be assigned to loss of a CH(CH_3_)_2_OH alcohol neutral unit, formed by rupture of the cyclohexanol ring accompanied by a hydrogen shift. The observation of this channel is consistent with the cyclohexanol oxygen being the protonation site. Finally, the figure illustrates pathway (4c) which is associated with production of fragment ion *m*/*z* 193.12. We assign this to the rupture of the cyclohexenyl ring, with partitioning of the excess charge onto the aromatic ring. This fragment is most easily rationalized by the proton being accommodated on the aromatic ring side of the molecule, in contrast to the expected protonation site that produces pathway (4b). The observation of fragmentation pathways associated with different protonation sites indicates that the precursor ion ensemble may consist of a combination of protonation isomers [29,30].

The HCD data for [THCOOH + H]^+^ are shown in Figure 3b, illustrating that the precursor ion fragments readily above 5% HCD energy to produce the *m*/*z* 327.19 fragment ion (formed upon loss of water) which reaches a maximum production intensity at 24% HCD. This behavior mirrors that of [THCOH + H]^+^ which also shows facile loss of water from the precursor ion with a very similar energy production profile. Above 10% HCD energy, a second relatively low-energy fragmentation channel is evident for [THCOOH + H]^+^, associated with production of the *m*/*z* 299.12 fragment ion. At higher collisional energies (>20% HCD energy), strong *m*/*z* 193.12 fragment ion channel is observed, as for [THCOH + H]^+^. At even higher collisional energies (>25% HCD energy), a group of lower-mass, secondary fragments are observed including *m*/*z* 123.04, 93.06, 91.05, and 79.05. This indicates that numerous fragmentation pathways are available at very high internal ion energies.

**Figure 5:**
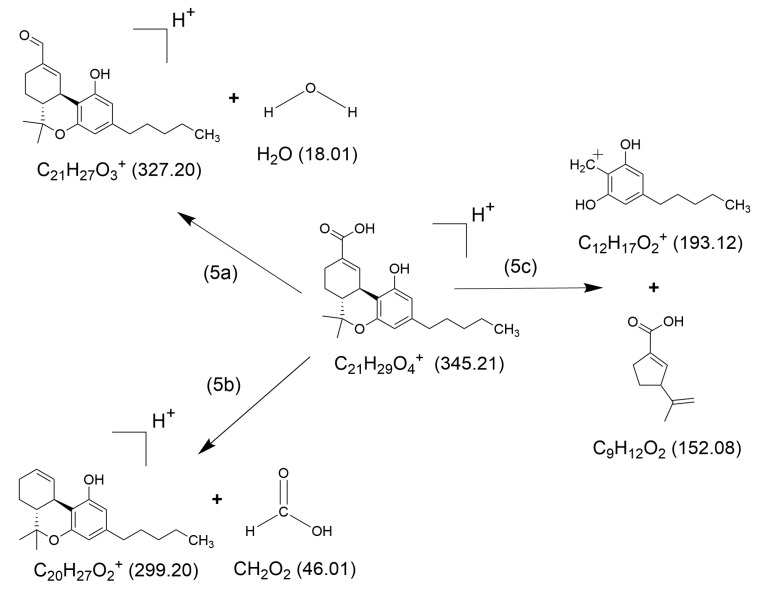
Suggested structures of the major ionic fragments and their neutral counter-fragments (5a)–(5c) observed in HCD experiment of [THCOOH + H]^+^. (Exact masses are given in parentheses).

The assigned major fragmentation pathways for [THCOOH + H]^+^ are displayed in Figure 5. The dominant low-energy HCD pathway, corresponding to loss of water, is shown in (5a). Pathway (5b) represents loss of a neutral formic acid unit with production of the *m*/*z* 299.12 fragment ion. Finally, *m*/*z* 193.12 production is shown in (5c) and is assigned to the rupture of the cyclohexanol ring, with ejection of the neutral pentenyl ring fragment. It should be noted that pathway (5c) only occurs above modestly high fragmentation energies and therefore has a significantly higher barrier than (5a) and (5b).

**Figure 6:**
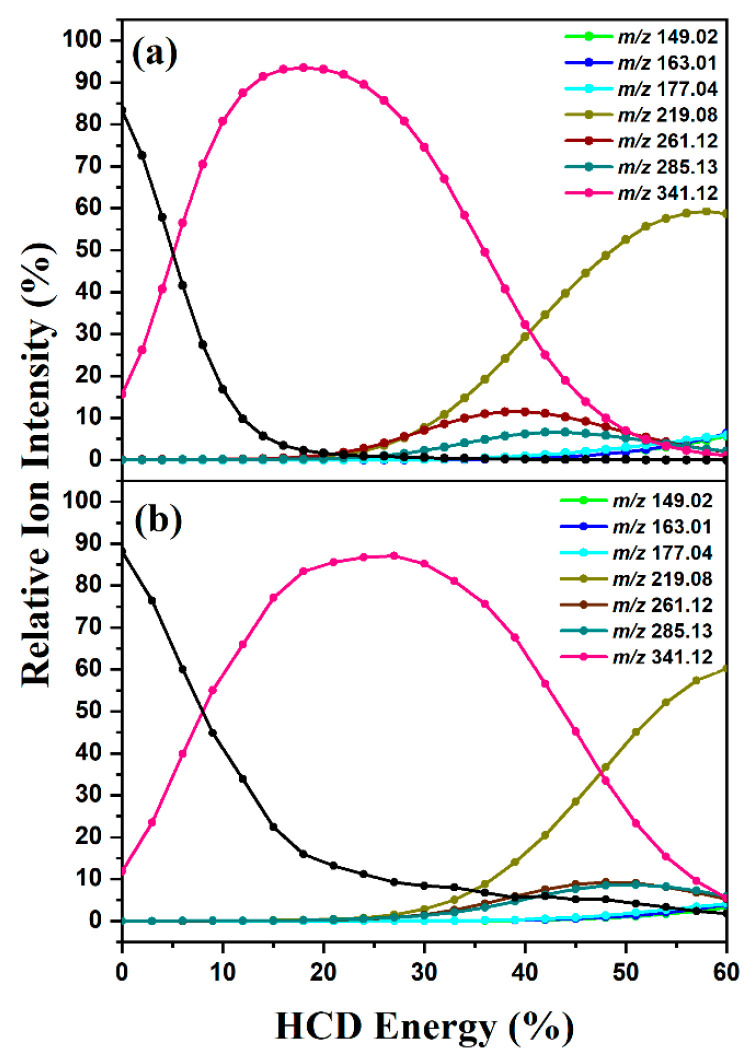
Precursor ion dissociation profile displayed with fragment ion production curves for (**a**) [CBDA + H]^+^ (*m*/*z* 359.18) and (**b**) [THCA + H]^+^ (*m*/*z* 359.18) displayed as a function of HCD energy. The solid line is a three-point adjacent average of the data points. The black line corresponds to the precursor ion.

Figure 6a shows the HCD fragmentation profile of [CBDA + H]^+^, illustrating that this molecular ion also fragments readily at very low collision energies. Indeed, for this ion, fragmentation is evident even at 0% HCD energy, indicating that the precursor ion is metastable upon isolation in the ion trap. The initial fragmentation of [CBDA + H]^+^ is associated with production of the dominant fragment ion, *m*/*z* 341.12, again associated with loss of water. Production of *m*/*z* 341.12 peaks at around 22% HCD energy. Beyond this energy, the intensity of the primary fragment decreases concomitant with the appearance of a number of secondary fragments, the most intense of which is the *m*/*z* 219.08 ion. The *m*/*z* 261.12 and *m*/*z* 285.13 ions are also significant intensity secondary fragments.

Before assigning the fragmentation pathways for [CBDA + H]^+^, it is instructive to inspect the HCD data for [THCA + H]^+^ (Figure 6b) and compare them to those of the isobaric ion [CBDA + H]^+^. Overall, the fragmentation profile is very similar but clearly not identical as was the case for the [CBD + H]^+^ and [THC + H]^+^ pair (Figure 1). While [THCA + H]^+^ is also metastable, like [CBDA + H]^+^, the dominant water loss fragmentation channel (*m*/*z* 341.22) peaks at a noticeably higher HCD energy of 27% and drops off less sharply towards the highest energies. Also, while the same group of fragment ions are observed for both precursors, the relative fragment ion intensities are not identical for [CBDA + H]^+^ and [THCA + H]^+^. The simplest explanation of these observations is that the barrier for interconversion between these precursor ions is low, so that tautomerization occurs as part of the collisional excitation process and results in the same group of fragment ions albeit at modestly different HCD energies. Since the [THCA + H]^+^ dissociation occurs at slightly higher energies, it can be concluded that this closed-ring structure lies energetically below that of the open-ring [CBDA + H]^+^.

**Figure 7:**
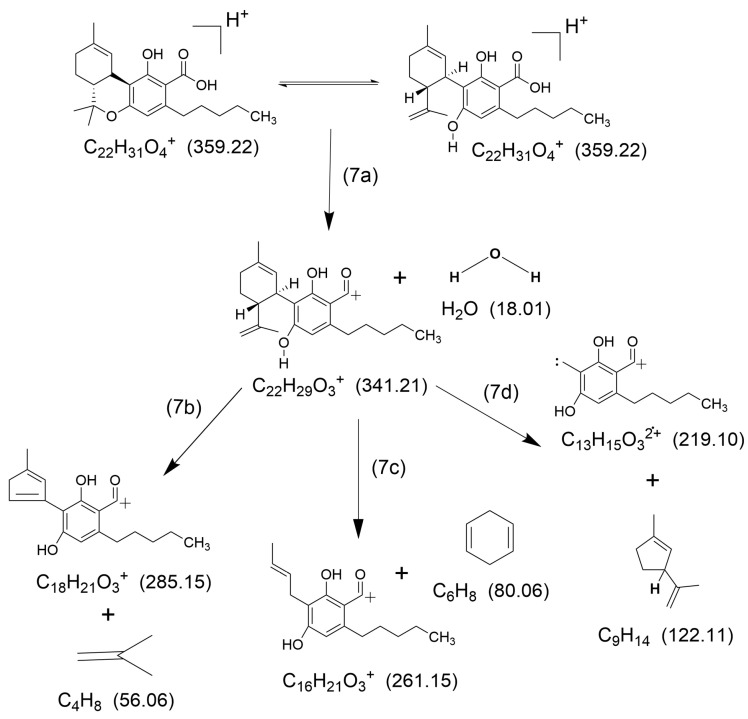
Suggested structures of the major ionic fragments and their neutral counter-fragments (7a)–(7d) observed in HCD fragmentation of [CBDA + H]^+^ and [THCA + H]^+^. The fragmentation pathways are illustrated for the [CBDA + H]^+^ molecular ion. Exact masses are given in parentheses.

Figure 7 presents an assignment of the likely fragmentation pathways of [CBDA + H]^+^ and [THCA + H]^+^. The two molecular ions are shown at the top of the figure, with the fragment ions being given for the [CBDA + H]^+^ open-ring tautomer. The dominant water loss fragmentation pathway is shown in (7a), leading to the dehydrated ion. Formation of the 285.15 amu fragment ion from the 341.21 amu precursor is depicted in (7b) showing loss of neutral butene. Pathway (7c) is associated with loss of a C_6_H_8_ neutral corresponding to a hexadienyl molecule. The final pathway depicted again corresponds to loss of the neutral pentenyl ring unit, which is also seen in [CBD + H]^+^, e.g., pathway (2a) in Figure 2 for loss of the neutral versus loss of the protonated species (2d). For [THCA + H]^+^, the charge remains with the 219.10 amu ion since it is located on the distant carbonyl group.

**Figure 8:**
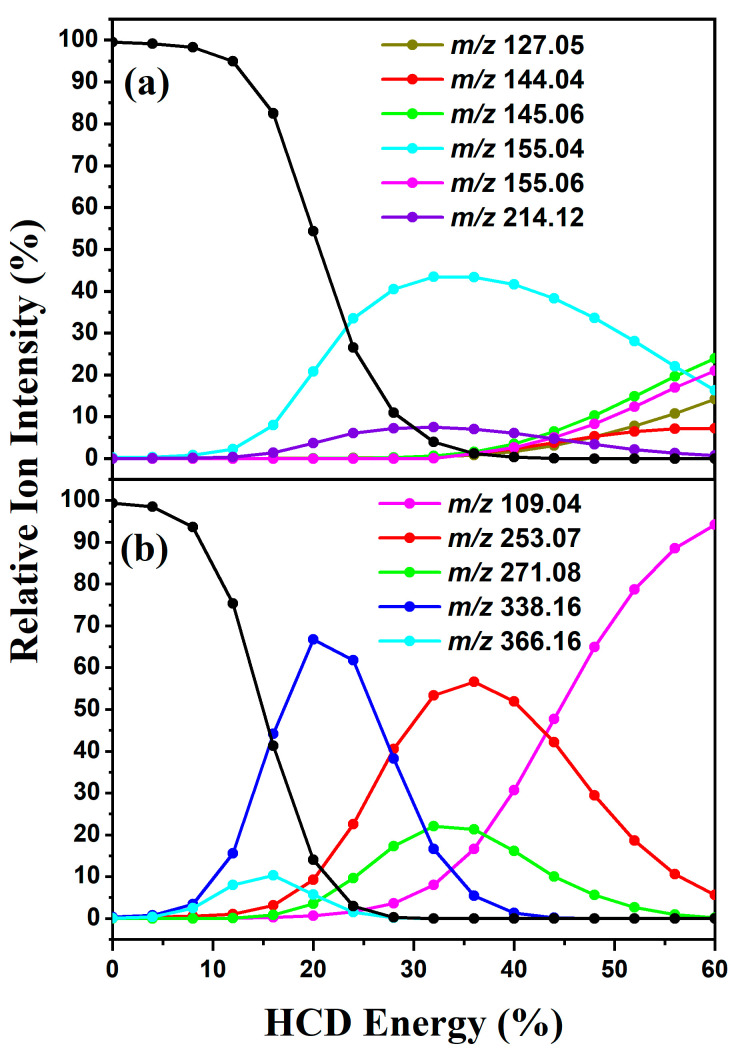
Precursor ion dissociation curve along with fragment ion production curves for (**a**) [JWH-018 + H]^+^ (*m*/*z* 342.18) and (**b**) [MDMB-FUBINACA + H]^+^ (*m*/*z* 398.18) displayed as a function of HCD energy. The solid line is a three-point adjacent average of the data points. The black line corresponds to the precursor ion.

**Figure 9:**
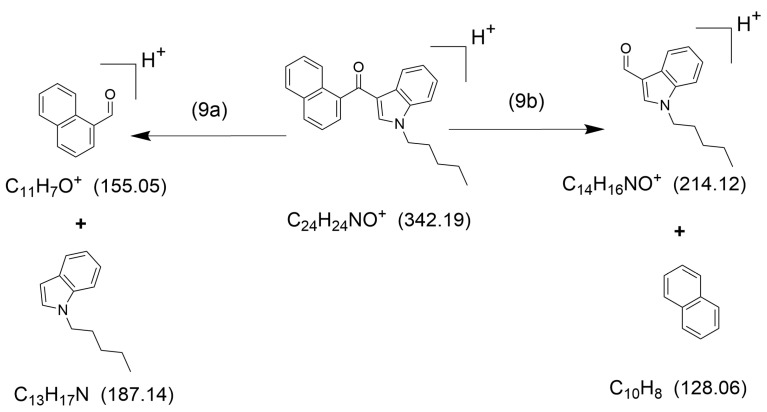
Suggested structures of the major ionic fragments and their neutral counter-fragments (9a)–(9b) observed in HCD fragmentation of [JWH-018 + H]^+^. Exact masses are given in parentheses.

Lastly, we present the HCD measurements for the synthetic cannabinoids, JWH-018 and MDMB-FUBINACA. Figure 8a displays the HCD fragmentation curves for [JWH-018 + H]^+^, illustrating that the precursor ion is stable at low HCD energies, and begins to fragment above ~12% HCD energy. The highest mass fragment produced is *m*/*z* 214.12, although the most intense fragment ion is *m*/*z* 155.04. Both of these ions are produced from above 12% HCD energy, with *m*/*z* 214.12 production peaking at 30% while that of *m*/*z* 155.04 peaks at a slightly higher value of HCD 35%. These two fragment ions arise from the two possible bond breaks at the central carbonyl group, i.e., pathways (9a) or (9b) in Figure 9. At higher energies, the intensities of the *m*/*z* 155.04 and *m*/*z* 214.12 pair of primary fragments decrease while a group of secondary fragments, *m*/*z* 127.05, 144.04, 145.06 and 155.06 grow in intensity as a function of collisional energy.

Xing et al. conducted GC-MS/MS experiments on [JWH-018 + H]^+^ using Collisional Dissociation in a triple quadrupole [3], and observed most of the fragments observed here. While *m*/*z* 145.06 and 155.06 were not observed in this previous work, they are only observed at the highest collisional energies in our work, indicating that the collisional energies employed by Xing et al. were below the threshold for producing these ions.

The HCD data for [MDMB-FUBINACA + H]^+^ are presented in Figure 8b, illustrating the molecular ion fragments from a relatively low-onset HCD energy (5%). Figure 8b shows that the primary fragments for [MDMB-FUBINACA + H]^+^ are the *m*/*z* 366.16 and 338.16 ions, with production profiles that peak close to 15 and 20% HCD energy, respectively. These ions can be assigned to loss of neutral methanol and HCO_2_CH_3_ molecules from the precursor ion (pathways 10a and 10b, respectively), indicating that fragmentation from the ester end group is facile.

**Figure 10:**
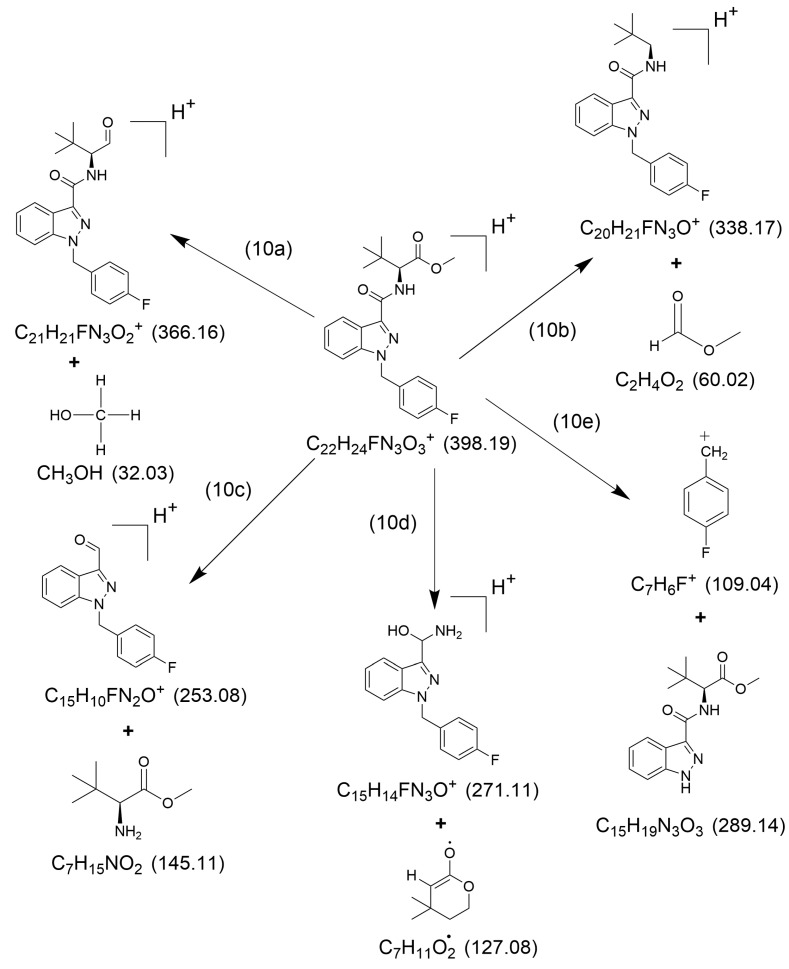
Suggested structures of the major ionic fragments and their neutral counter-fragments (10a)–(10e) observed in HCD fragmentation of [MDMB-FUBINACA + H]^+^. Exact masses are given in parentheses.

The next pair of fragment ions, *m*/*z* 253.07 and 271.08, displays moderately different production maxima of 34% and 37% HCD energy, respectively. These fragments can be assigned to rupture on either side of the amide N atom, i.e., pathways (10c) and (10d) in Figure 10. Above 44% HCD energy, production of a *m*/*z* = 109.05 fragment increases strongly due to the opening of a new high-internal-energy dissociation pathway, which corresponds to rupture of the C-N bond adjacent to the fluorine-containing aromatic group (10e).

### 2.2. Quantum Chemistry Calculations

To our knowledge, the geometric structures of the protonated cannabinoid molecules studied in this work have not been investigated using computational chemistry. We performed preliminary calculations on the [CBDA + H]^+^ and [THCA + H]^+^ pair of tautomers as part of this work to explore the preferred protonation sites and geometric structures. We note that these molecules contain considerable confirmation flexibility, which should be explored more extensively in future work, since tautomeric and conformational energies can be competitive [31].

**Figure 11:**
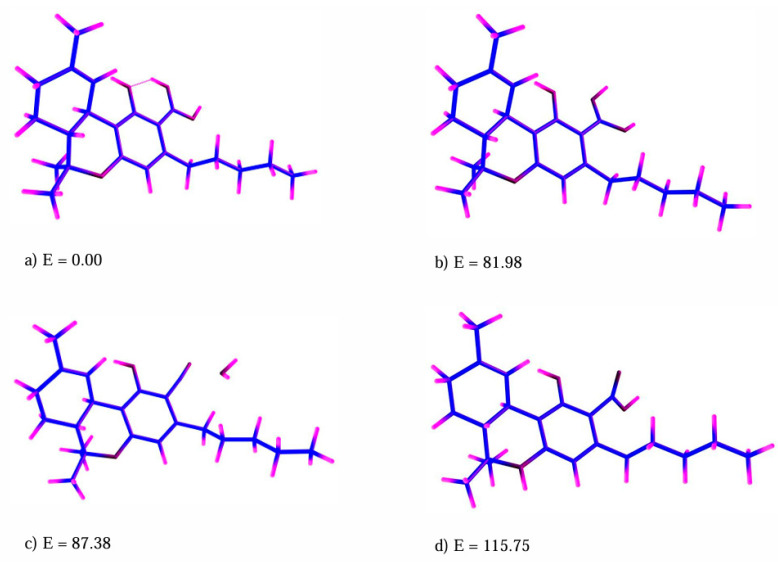
Four low-energy geometric structures of [THCA + H]^+^, obtained at M062X/6-311G (d,p) level of theory. (**a**) shows the global minimum structure identified, (**b**)–(**d**) show higher energy structures. Relative energies (in kJ/mol) are included with the structures.

Figure 11 displays four low-energy structures of [THCA + H]^+^ calculated at the M062X/6-311G (d,p) level of theory. Relative energies (in kJ/mol) are included in the figure. From the structures we explored, the lowest-energy structure obtained (Figure 11a) corresponds to a protonation isomer where protonation has occurred on the carbonyl of the carboxylic acid group. Notably, this structure benefits from the presence of an intramolecular hydrogen bond between two adjacent phenol OH groups. The similar structure (a rotational isomer) where this intramolecular hydrogen bond is broken (Figure 11b) is 81.98 kJ/mol higher in energy than the lowest-energy structure. Figure 11c displays a geometric structure where a water molecule has dissociated from [THCA + H]^+^ illustrating that water loss from this system is effectively barrierless. The water molecule is hydrogen-bonded to the remaining molecular fragment in this minimum-energy structure. This is consistent with the experimental results (Figure 7) which showed that the cation was metastable with respect to water loss (Pathway 7a). Finally, Figure 11d displays a protonation isomer where the cyclohexenyl oxygen is protonated. Although the structure displayed is 115.75 kJ/mol higher in energy than the global minimum structure (Figure 11a), it does not contain an intramolecular hydrogen bond, which accounts for ~80 kJ/mol of this energy difference. Nonetheless, this still clearly indicates that protonation at the carboxylic acid site is calculated to be energetically more favorable than protonation on the cyclohexenyl oxygen.

**Figure 12:**
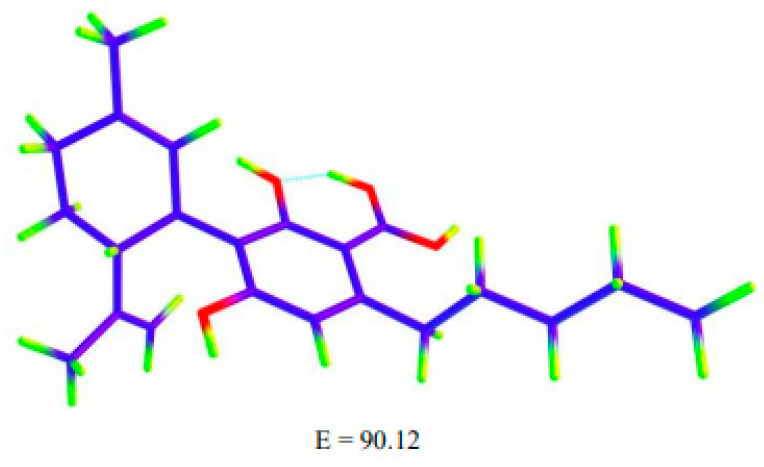
A low-energy geometric structure of [CBDA + H]^+^, obtained at M062X/6-311G (d,p) level of theory, which is comparable to the lowest-energy structure of [THCA + H]^+^, (Figure 11a). This structure is 90.12 kJ/mol above that of the [THCA + H]^+^ structure shown in Figure 11a.

[CBDA + H]^+^ structures were also explored by computational chemistry. Figure 12 displays a low-energy geometric structure of [CBDA + H]^+^, where protonation occurs at the carboxylic acid group and the phenol OH groups adopt an intramolecular bond as in the lowest-energy isomer of [THCA + H]^+^ (Figure 11a). This [CBDA + H]^+^ structure lies 90.12 kJ/mol higher in energy compared to the structure in Figure 11a, illustrating that the closed-ring [THCA + H]^+^ tautomeric structure is lower in energy than the open-ring [CBDA + H]^+^ one. Indeed, in our wider optimization of structures, we observed several instances where [CBDA + H]^+^ open-ring starting structures collapsed to closed-ring [THCA + H]^+^ ones.

The quantum chemical calculations performed therefore show that under protonation conditions, the closed-ring [THCA + H]^+^ geometric structure is energetically preferred over the open-ring one [CBDA + H]^+^. In addition, they confirm that protonation preferentially occurs on the carboxylic acid group, and a water molecule can be readily lost (energetically) from this protonation isomer. We anticipate that the quantum chemical results for the isobaric pair, [CBDA + H]^+^ and [THCA + H]^+^, can be extended to the other isobaric pair of ions studied here, namely [CBD + H]^+^ and [THC + H]^+^, thus explaining the similar HCD profiles for these ions (Figure 2) as arising from isomerization of the open-ring [CBD + H]^+^ into a lower-energy closed-ring [THC + H]^+^ structure.

## 3. Discussion

The HCD measurements for the naturally occurring cannabinoids presented above allow us to identify some common fragmentation patterns. Firstly, for molecules that contain carboxylic acid groups (THCOOH, THCA and CBDA), protonation appears to occur preferentially on the carboxylic acid, and loss of a neutral water molecule is a very low-energy fragmentation pathway. Indeed, [THCA + H]^+^ and [CBDA + H]^+^ are metastable with respect to water loss. Other fragmentation pathways associated with production of a stable neutral molecule are common for all of the protonated natural cannabinoids, e.g., fragmentation pathway 5b for [THCOOH + H]^+^ which results in ejection of neutral formic acid, and pathway 2c for [CBD + H]^+^ where loss of butene occurs.

A second common fragmentation motif for the protonated natural cannabinoids involves break-up of the molecular ion by fission of the central C-C bond that links the aromatic and alkyl ring groups, e.g., pathway 2a for [CBD + H]^+^. Several such fragmentation pathways are associated with β cleavage and production of a pentenyl ring fragment, e.g., pathway 7d for [CBDA + H]^+^/[THCA + H]^+^. This appears to be an especially characteristic fragmentation motif for protonated cannabinoid systems.

The two synthetic cannabinoids again display dominant fragmentation pathways that are associated with ejection of stable neutral molecules, e.g., pathway (9b) of [JWH-018 + H]^+^ which includes loss of neutral naphthalene. Aside from this commonality, the pathways of the two synthetic cannabinoids are distinctive, and reflect their strikingly different molecular structures. This leads to the conclusion that while the fragmentation pathways of the natural cannabinoids could be used in a predictive fashion for other natural cannabinoids and metabolites, this would be of limited value for synthetic cannabinoids due to their considerable structural diversity.

Aside from their value in predicting fragmentation pathways for similar molecules, the HCD measurements presented are useful in a number of additional ways. For example, an understanding of metastable breakdown pathways for a precursor ion is important in conducting suspect-screening measurements, since the intensity of a precursor ion in a mass spectrum may be significantly lower than that of the breakdown product ion. In such a case, it would be preferential to suspect-screen for the breakdown product ion rather than the precursor ion [32]. Energy-resolved HCD measurements are also important for interpreting laser photodissociation measurements since they provide insight into primary and secondary photoproducts [33,34].

## 4. Materials and Methods

**General.** Cannabidiol (CBD), Δ9-tetrahydrocannabinol (THC), 11-nor-9-carboxy-Δ9-tetrahydrocannabinol (THC-OOH) and 11-hydroxy-Δ9-tetrahydrocannabinol (THC-OH) standards were purchased from Cambridge Bioscience (Cambridge, UK), while cannabidiolic acid (CBDA), tetrahydrocannabinolic acid (2-COOH-THC), MDMB-FUBINACA and JWH-018, were purchased from Sigma Aldrich (Gillingham, UK). All standards were purchased as solutions in methanol except for MDMB-FUBINACA and Tetrahydrocannabinolic acid, which were in acetonitrile. For electrospray, all of the commercial cannabinoid samples were diluted to 1 × 10^−5^ M in either methanol or acetonitrile as appropriate for the compound being studied (i.e., compounds that were purchased as a solution in methanol were diluted in methanol, while compounds purchased as solutions in acetonitrile were diluted in acetonitrile). HPLC-grade methanol and acetonitrile were used to dilute the standards and were purchased from Fisher Scientific, Inc. (Pittsburgh, PA, USA).

**Collision-Induced Dissociation**. Higher-energy Collisional Dissociation (HCD) was performed to investigate the ground-state thermal fragmentation characteristics of [CBD + H]^+^, [THC + H]^+^, [THCOH + H]^+^, [THCOOH + H]^+^, [CBDA + H]^+^, [THCA + H]^+^, [JWH-018 + H]^+^, and [MDMB-FUBINACA + H]^+^ on an Orbitrap™ Fusion Tribrid mass spectrometer (Thermo Fisher Scientific, Waltham, MA, USA) with an ESI source described previously [24,35]. Experiments were performed in positive ion mode between 0% and 60% collisional energy at a flow rate of 3.0 µL/min, with the following parameters: MS2 scan isolation mode, ion trap; detector type, ion trap; ion spray voltage (3500 V); RF lens (60%); normalized AGC target (100%); maximum injection time (100 ms); ion transfer tube temperature (280 °C); and vaporizer temperature (20 °C). For the MS scan in this instrument, the settings were as follows: detector type, Orbitrap; positive ion spray voltage (3200 V); RF lens (45%); normalized AGC target (100%); and maximum injection time (100 ms). All experiments were performed in triplicates and the averages are used for data presented. Errors between different repeat measurements were within ±3%. Positive ion mode is generally preferred given that more compounds ionize in the positive mode when liquid chromatography is coupled to an electrospray ion source mass spectrometer [36,37].

**Quantum Chemical Calculations**: Density functional theory calculations were conducted at the M062X/6-311++G (2d, 2p) level of theory using Gaussian 16 on a selection of geometric structures of the tautomeric pair of molecular ions, [CBDA+ H]^+^ and [THCA + H]^+^ [38]. A range of starting geometric structures were obtained using Gaussview 6 (both conformational isomers and protonation isomers), with energy smoothing conducted on the initial structures within Gaussview 6. Frequency calculations were conducted on the optimized structures to ensure that they correspond to true energy minima.

## 5. Conclusions

Higher Collisional Dissociation (HCD) was performed on a range of natural and synthetic cannabinoids to measure the energy-resolved fragmentation pathways. The measurements allowed the identification of a range of common fragmentation pathways for the natural cannabinoids, which could be used in a predictive manner for similar molecular systems. Quantum chemical calculations were performed on one of the tautomeric pairs of cannabinoids (i.e., [CBDA + H]^+^ and [THCA + H]^+^). The calculations indicated that the closed-ring form of [THCA + H]^+^ was the preferred tautomeric form, with protonation occurring on the carboxylic acid group. Although a number of open-ring starting optimizations collapsed to closed-ring structures, two distinctive global minima structures were obtained for [CBDA + H]^+^ and [THCA + H]^+^, consistent with a non-zero barrier being present between these two tautomers.

One of the key findings from the energy-dependent fragmentation profiles obtained is their value in unambiguously identifying systems where tautomerization has occurred prior to or during ionization. We found that the [THC + H]^+^ and [CBD + H]^+^ isobaric pair displayed identical energy-dependent fragmentation profiles, indicating that tautomerization occurs from one system to the other, as has previously been suggested [39,40,41,42]. For the isobaric [CBDA + H]^+^ and [THCA + H]^+^ pair, the energy-dependent fragmentation profiles for the two molecular ions were highly similar, but importantly not identical, as was the case for [THC + H]^+^ and [CBD + H]^+^. This indicated that a small energy barrier exists between the two, with the closed-ring [THCA + H]^+^ fragmenting at very slightly higher HCD energy, consistent with this structure being the global minimum structure. It is notable that this conclusion is entirely consistent with the quantum chemical calculations. More generally, the results show that energy-dependent fragmentation profiles have utility for characterizing tautomeric ions, both in terms of identifying cases where tautomerization from one structure to another is spontaneous, but also for establishing cases where a barrier exists between one tautomeric form and another.

## Data Availability

Data are available from the corresponding author upon request.
